# Supplementary material for: Models optimized for real-world tasks reveal the task-dependent necessity of precise temporal coding in hearing
Source: Nat Commun. 2024 Dec 4;15:10590. doi: 10.1038/s41467-024-54700-5 (PMC11618365; doi:10.1038/s41467-024-54700-5)
Supplement: Supplementary file 1 — Supplementary Information [file 41467_2024_54700_MOESM1_ESM.pdf]

SUPPLEMENTARY INFORMATION

Models optimized for real-world tasks reveal the task-dependent necessity of precise temporal coding in hearing

Mark R. Saddler & Josh H. McDermott

TABLE OF CONTENTS

1. **Supplementary Fig. 1** | Models with access to phase-locked spike timing have better and more human-like hearing (alternate human-model similarity metric)
2. **Supplementary Fig. 2** | Effect of phase locking on all sound localization experiments
3. **Supplementary Fig. 3** | Localization models with degraded auditory nerve spike timing rely on spectral cues to judge azimuth as well as elevation
4. **Supplementary Fig. 4** | Models optimized separately for word and voice recognition -- effect of phase locking on all speech experiments
5. **Supplementary Fig. 5** | Models optimized jointly for word and voice recognition -- effect of phase locking on all speech experiments
6. **Supplementary Fig. 6** | Word and voice recognition in real-world auditory textures
7. **Supplementary Fig. 7** | Model word and voice recognition with inharmonic speech in noise
8. **Supplementary Fig. 8** | Models optimized jointly for word and voice recognition exhibit a larger effect of tone vocoding than models optimized solely for word recognition
9. **Supplementary Fig. 9** | Comparison of model results with detailed vs. simplified cochlear stages
10. **Supplementary Fig. 10** | Simplified cochlear model -- effect of phase locking on all sound localization experiments
11. **Supplementary Fig. 11** | Simplified cochlear model -- effect of phase locking on all speech experiments.
12. **Supplementary Fig. 12** | Effect of increasing auditory nerve sampling rate from 10 to 20 kHz
13. **Supplementary Fig. 13** | Effect of increasing auditory nerve sampling rate from 10 to 20 kHz on all sound localization experiments
14. **Supplementary Fig. 14** | Effect of increasing auditory nerve sampling rate from 10 to 20 kHz on all speech experiments
15. **Supplementary Table 1** | Neural network architectures for sound localization models
16. **Supplementary Table 2** | Neural network architectures for word and voice recognition models
17. **Supplementary Table 3** | Neural network architectures for frequency discrimination models
18. **Supplementary References**

Source data and code to generate all main text and supplementary figures are available at https://github.com/msaddler/phaselocknet.

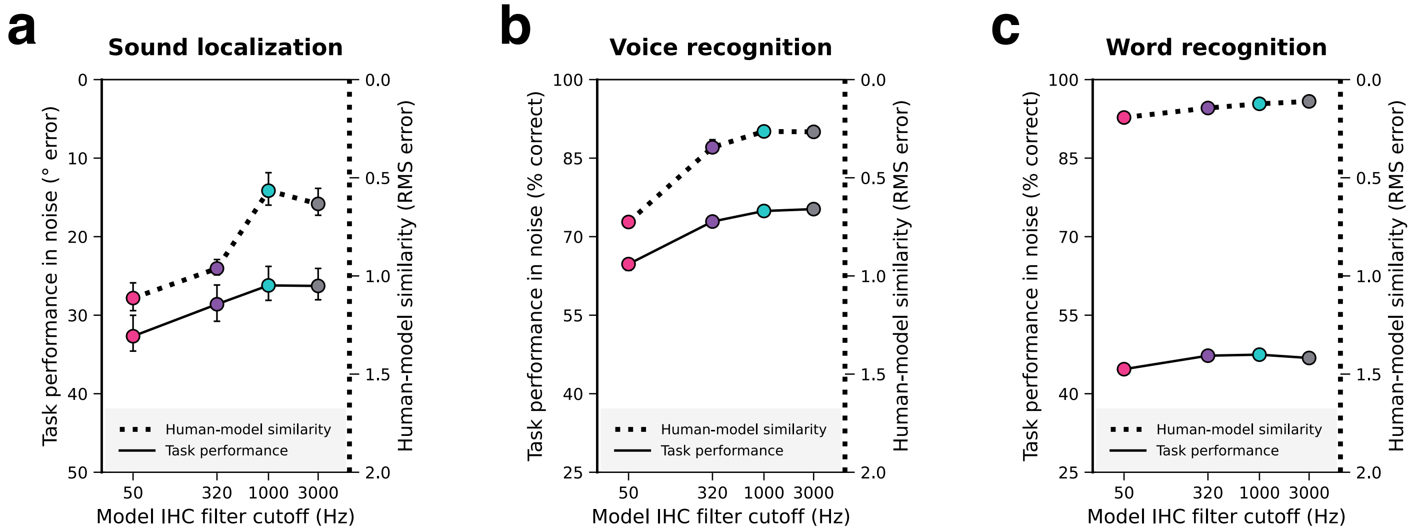

Supplementary Fig. 1 I Models with access to phase-locked spike timing have better and more human-like hearing (alternate human-model similarity metric). As in Fig. 2, each panel corresponds to a different task and summarizes the effect of auditory nerve phase locking limit on i) naturalistic model task performance and ii) overall human-model behavioral similarity. The difference from Fig. 2 is that overall human-model behavioral similarity is quantified as the root-mean-squared error between analogous human and model data points, min-max normalized by the human data to account for differences in measurement units across experiments and then averaged across all experiments for each model task (right y-axes; dotted lines). The right y-axes are inverted such that higher positions correspond to more human-like behavior. Naturalistic task performance is quantified as a single number averaged across noise conditions (left y-axes; solid lines). Error bars indicate 95% confidence intervals bootstrapped across 10 network architectures for each model. **a.** Sound localization. The left y-axis plots mean absolute error for the sound localization model and is inverted so that better model performance corresponds to higher positions on the y-axis. **b.** Voice recognition. Here and in **c,** the left y-axes plot percent correct for the model when tested on speech in noise. **c.** Word recognition. All models reproduced human word recognition fairly well according to this alternative metric, but the 50 Hz model was still worst overall, and the change in human-model similarity, while modest, was largest between the 50 Hz and 320 Hz models than between the other phase locking limits. We note that a model only needs to appear worse than others according to one metric to be ruled out, and the correlation metric was more diagnostic in this case. This is because the 50 Hz model exhibits a qualitative discrepancy for one experiment (Fig. 7a-c), and this is revealed most clearly with a correlation metric.

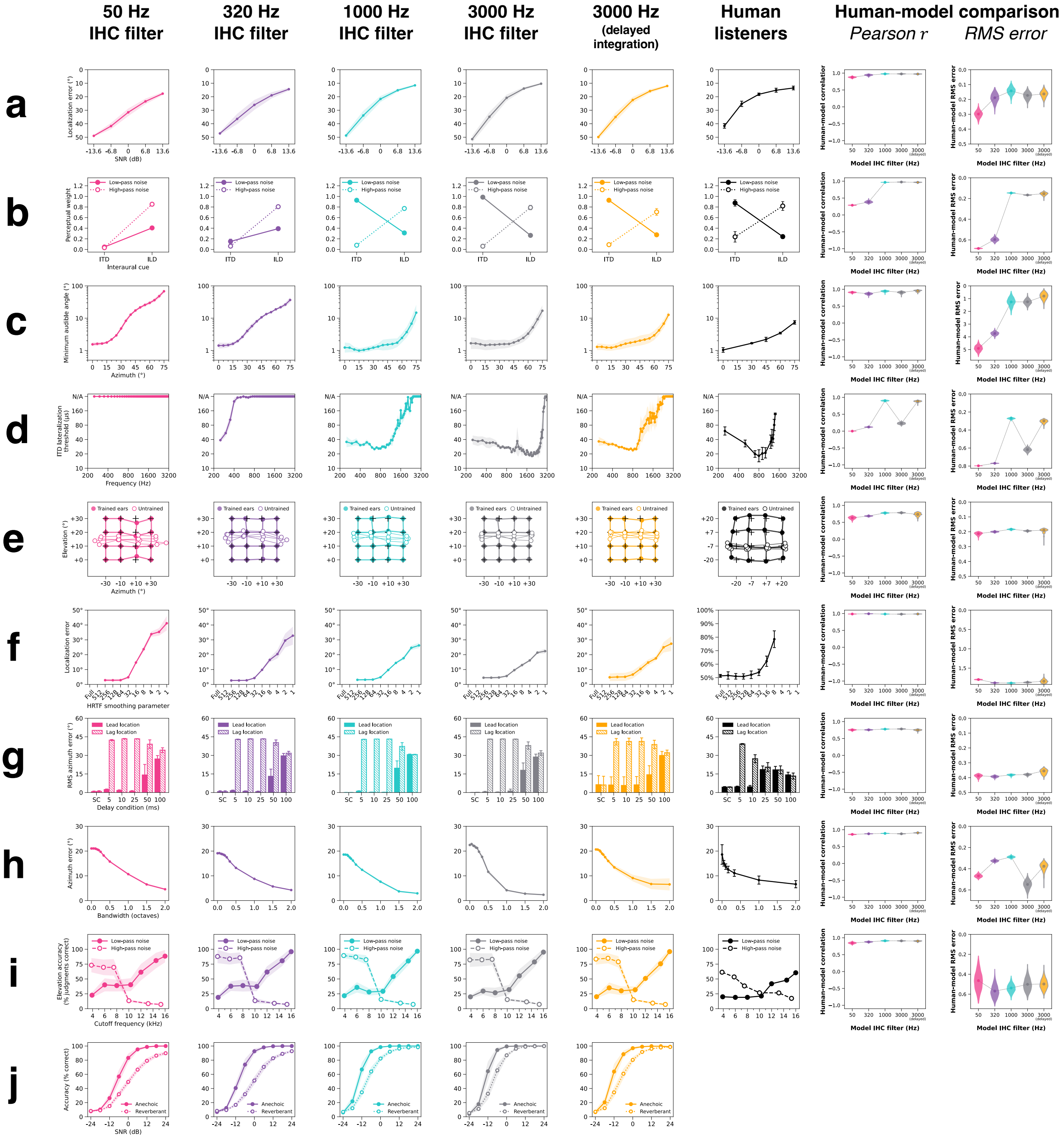

Supplementary Fig. 2 | Effect of phase locking on all sound localization experiments. This grid summarizes the behavioral data used to measure human-model similarity scores for the localization models. The first four columns correspond to models optimized with different phase locking limits. The fifth (orange) column corresponds to the 3000 Hz phase locking model with network architectures modified to delay binaural integration. The sixth column contains results from human listeners. The rightmost two columns quantify human-similarity by measuring Pearson correlations and root-mean-squared error between analogous human and model data points. Violin plots depict bootstrapped distributions of human-model similarity scores across 10 network architectures per phase locking condition. Rows correspond to 10 different sound localization experiments. **a.** Sound localization in noise. **b.** Minimum audible angle vs. frequency. **c.** ITD / ILD cue weighting. **d.** ITD lateralization vs. frequency. **e.** Effect of changing ears. **f.** Effect of smoothing spectral cues. **g.** Precedence effect. **h.** Bandwidth dependency of localization. **i.** Median plane spectral cues. **j.** Speech localization in noise and reverberation (model experiment only). All model error bars indicate ±2 standard errors of the mean across 10 network architectures.

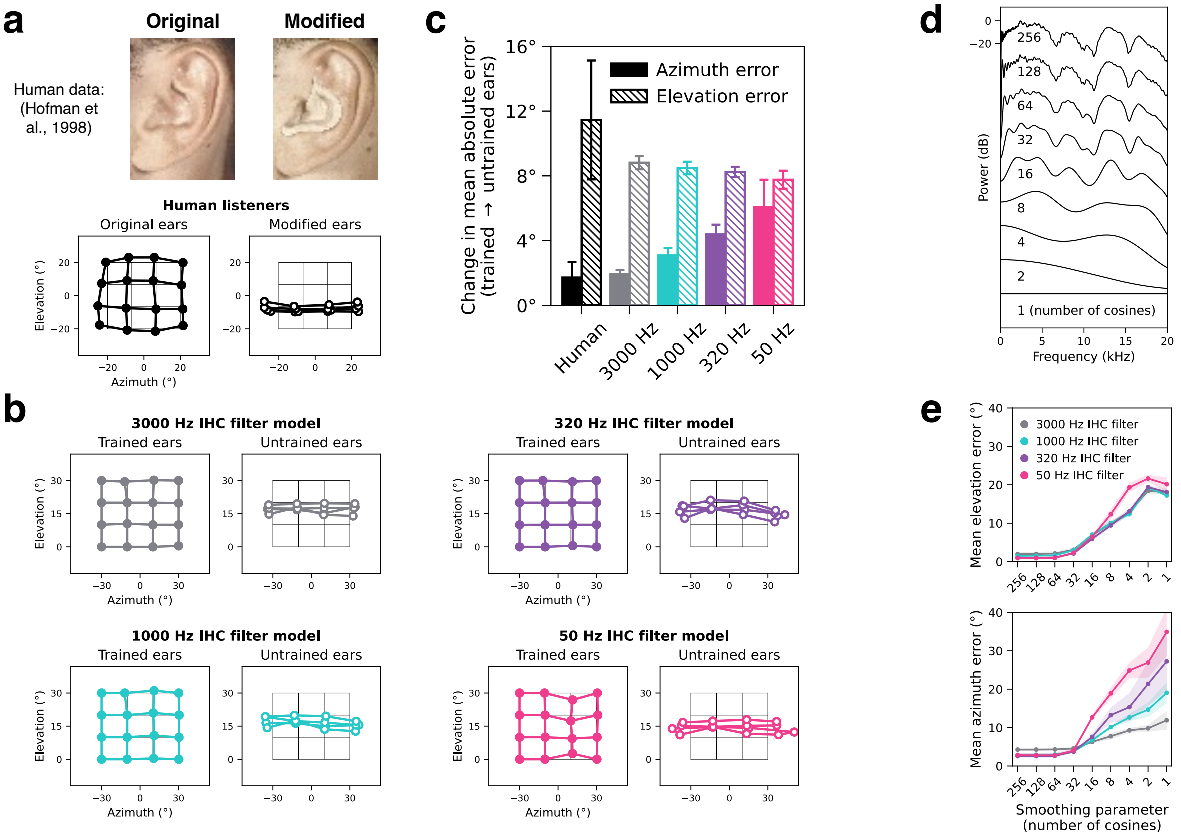

Supplementary Fig. 3 | Localization models with degraded auditory nerve spike timing rely on spectral cues to judge azimuth as well as elevation. Whereas localization in azimuth is dominated by binaural cues, localization in elevation is mediated in large part by spectral cues from the pinnae of the outer ear. Manipulating these spectral cues -- either physically by altering pinna shape with an ear mold[1] or virtually by altering the head-related transfer function (HRTF) used to spatialize a sound[2] via earphones -- impairs elevation judgments by humans. These same manipulations have minimal effects on azimuth judgments. This figure shows the results of altered phase locking limits on the effect of spectral cues to localization.

Hofman et al. (1998) measured human localization of white noise bursts before and after inserting plastic molds into participants' ears to change the pinnae's direction-specific filtering (**a**). Human sound localization judgments (thick lines, circle markers) with the participants' original (left) and modified (right) ears are plotted as a function of azimuth and elevation, superimposed on a grid of the true locations (thin lines, no markers). Data were scanned from the original study[1], averaged across 4 participants, and re-plotted here. Photographs in **a** reproduced with permission from Springer Nature: Hofman et al., Nature Neuroscience, Volume 1 no 5, page 418, September 1998, Springer Nature. In an analogous experiment (**b**), we evaluated models with four different phase locking limits on white noise bursts rendered with either the HRTFs used for training (trained ears) or a different set of HRTFs (untrained ears). Models were always trained using HRTFs measured from a standard model of the human head and torso[3] (KEMAR). The model "untrained ears" were alterative HRTFs measured from the ears of 45 different people (results shown are averaged across the 45 sets of HRTFs). Model data are plotted with the same conventions as in (**a**). When tested with alternative pinnae, elevation judgments collapsed in all models, as in human listeners with ear molds, indicating that spectral cues were used irrespective of phase locking. However, the effect of alternative ears on azimuth was different for different phase locking cutoffs. Panel (**c**) plots the increase in mean absolute azimuth and elevation error due to ear alteration for humans and for each model. Error bars indicate ±2 standard errors of the mean across 4 human participants or 10 network architectures. In human listeners and in models with high-fidelity temporal coding, changing pinnae had little effect on azimuthal accuracy. But in models with degraded temporal coding, azimuthal localization accuracy was worse with alternative pinnae indicating that the absence of phase locking rendered models dependent on pinna cues for azimuthal localization, unlike humans. These results suggest that human-like dependence on ear-specific cues (i.e., only for elevation) emerges only when models have access to phase-locked spike timing.

This non-human-like dependence of azimuthal localization on monaural spectral cues was also evident in the effects of removing spectral details from the cues. We progressively smoothed the power spectra of the trained HRTFs by lowering the number of cosines used to approximate the discrete cosine transform (**d**). We measured the effect of this spectral smoothing on model localization of white noise bursts. Mean absolute elevation (top) and azimuth (bottom) errors are plotted as a function of the HRTF smoothing parameter used to render stimuli for the models (**e**). Error bars indicate ±2 standard errors of the mean across 10 network architectures. As the peaks and valleys of the trained HRTFs were parametrically smoothed away, model elevation judgments progressively collapsed, regardless of phase locking limit, as expected. By contrast, azimuth judgments were significantly more impaired by the smoothing in models with lower phase locking limits, suggesting they learned to use fine spectral details to localize in azimuth as well as elevation (unlike humans with normal hearing but consistent with the behavior of some single-sided cochlear implant users[4]).

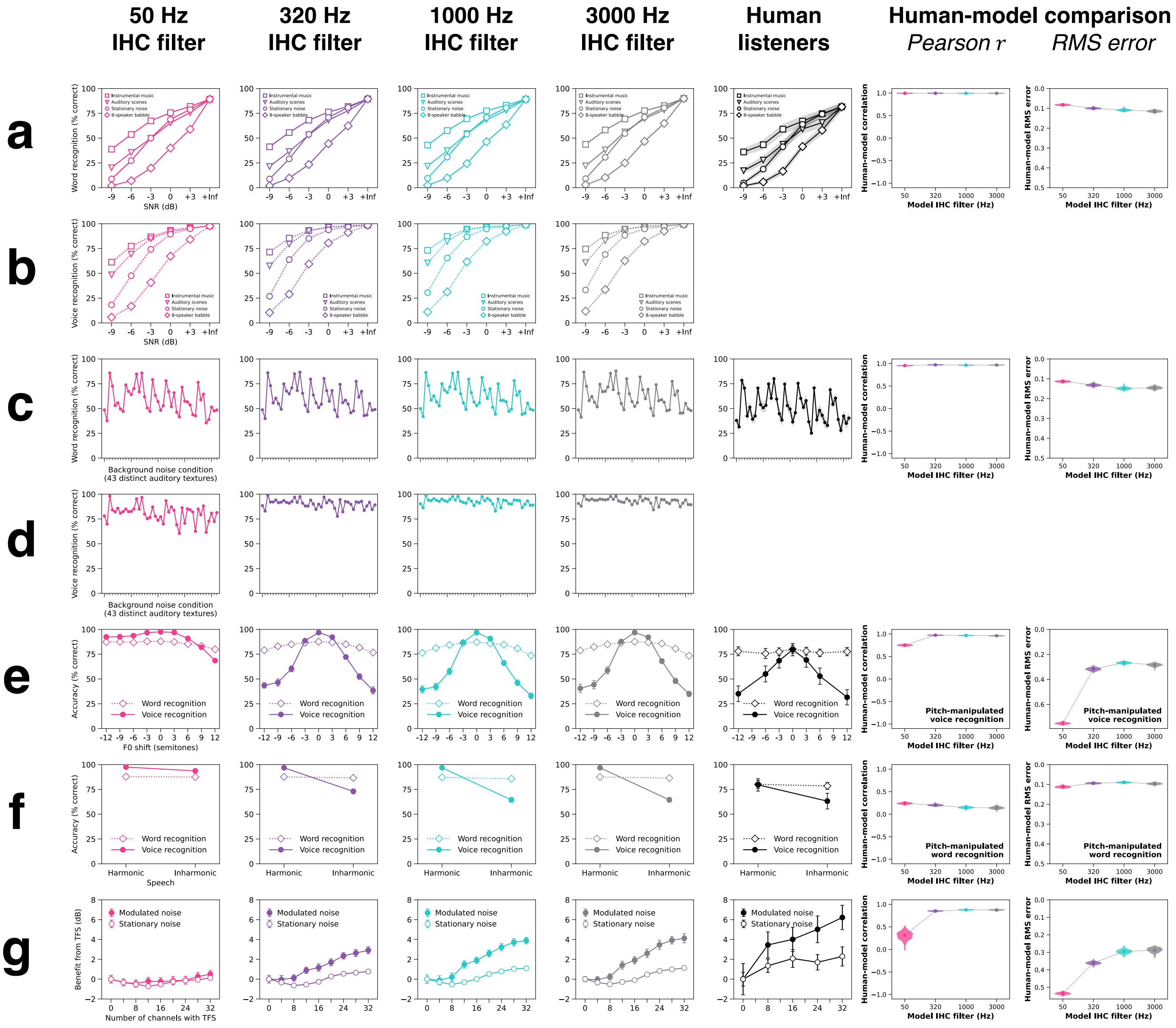

Supplementary Fig. 4 | Models optimized separately for word and voice recognition -- effect of phase locking on all speech experiments. The same network architectures optimized jointly for word and voice recognition in the main text were also optimized separately for the word and voice recognition tasks for each phase locking condition. This yielded similar results to the jointly optimized models. The first four columns correspond to models optimized with different phase locking limits. The fifth column contains results from human listeners. The rightmost two columns quantify human-similarity by measuring Pearson correlations and root-mean-squared error between analogous human and model data points. Violin plots depict bootstrapped distributions of human-model similarity scores across 10 network architectures per phase locking condition. Rows correspond to 7 speech experiments. **a.** Word recognition in real-world noise conditions. **b.** Voice recognition in real-world noise conditions (model experiment only). **c.** Word recognition in 43 distinct auditory textures at -3 dB SNR. **d.** Voice recognition in 43 distinct auditory textures at -3 dB SNR (model experiment only). **e.** Word and voice recognition with F0-shifted speech. **f.** Word and voice recognition with harmonic and inharmonic speech. **g.** Effect of tone vocoding on word recognition in stationary and modulated noise. All model error bars indicate ±2 standard errors of the mean across 10 network architectures.

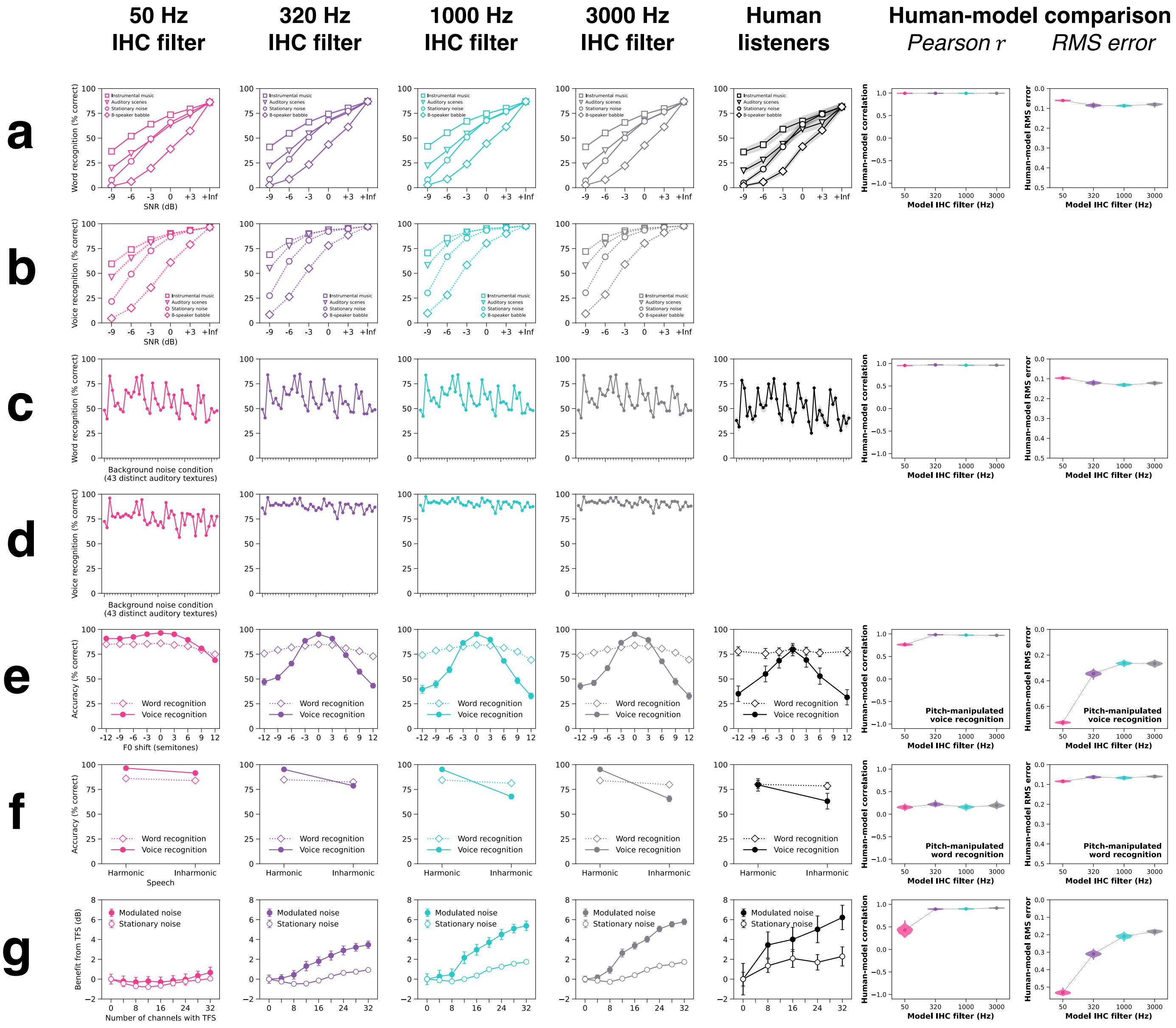

Supplementary Fig. 5 | Models optimized jointly for word and voice recognition -- effect of phase locking on all speech experiments. This grid summarizes the behavioral data used to measure human-model similarity scores for the word and voice recognition models. The first four columns correspond to models optimized with different phase locking limits. The fifth column contains results from human listeners. The rightmost two columns quantify human-similarity by measuring Pearson correlations and root-mean-squared error between analogous human and model data points. Violin plots depict bootstrapped distributions of human-model similarity scores across 10 network architectures per phase locking condition. Rows correspond to 7 speech experiments. **a.** Word recognition in real-world noise conditions. **b.** Voice recognition in real-world noise conditions (model experiment only). **c.** Word recognition in 43 distinct auditory textures at -3 dB SNR. **d.** Voice recognition in 43 distinct auditory textures at -3 dB SNR (model experiment only). **e.** Word and voice recognition with F0-shifted speech. **f.** Word and voice recognition with harmonic and inharmonic speech. **g.** Effect of tone vocoding on word recognition in stationary and modulated noise. All model error bars indicate ±2 standard errors of the mean across 10 network architectures.

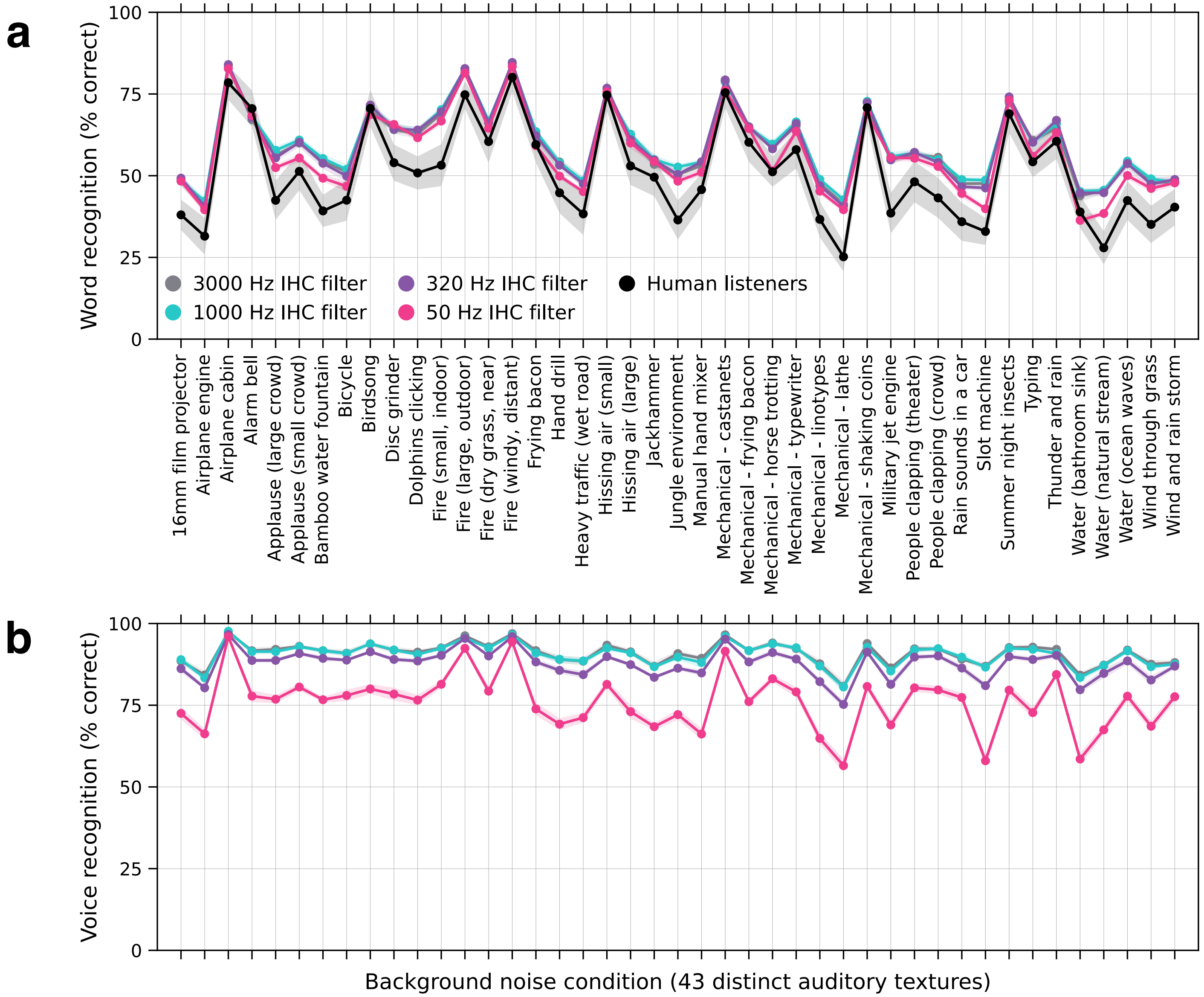

Supplementary Fig. 6 | Word and voice recognition in real-world auditory textures. a. Human and model word recognition for speech embedded in 43 distinct auditory textures at -3 dB SNR. **b.** Model voice recognition for the same stimuli. Error bars indicate ±2 standard errors of the mean across 47 human participants or 10 network architectures.

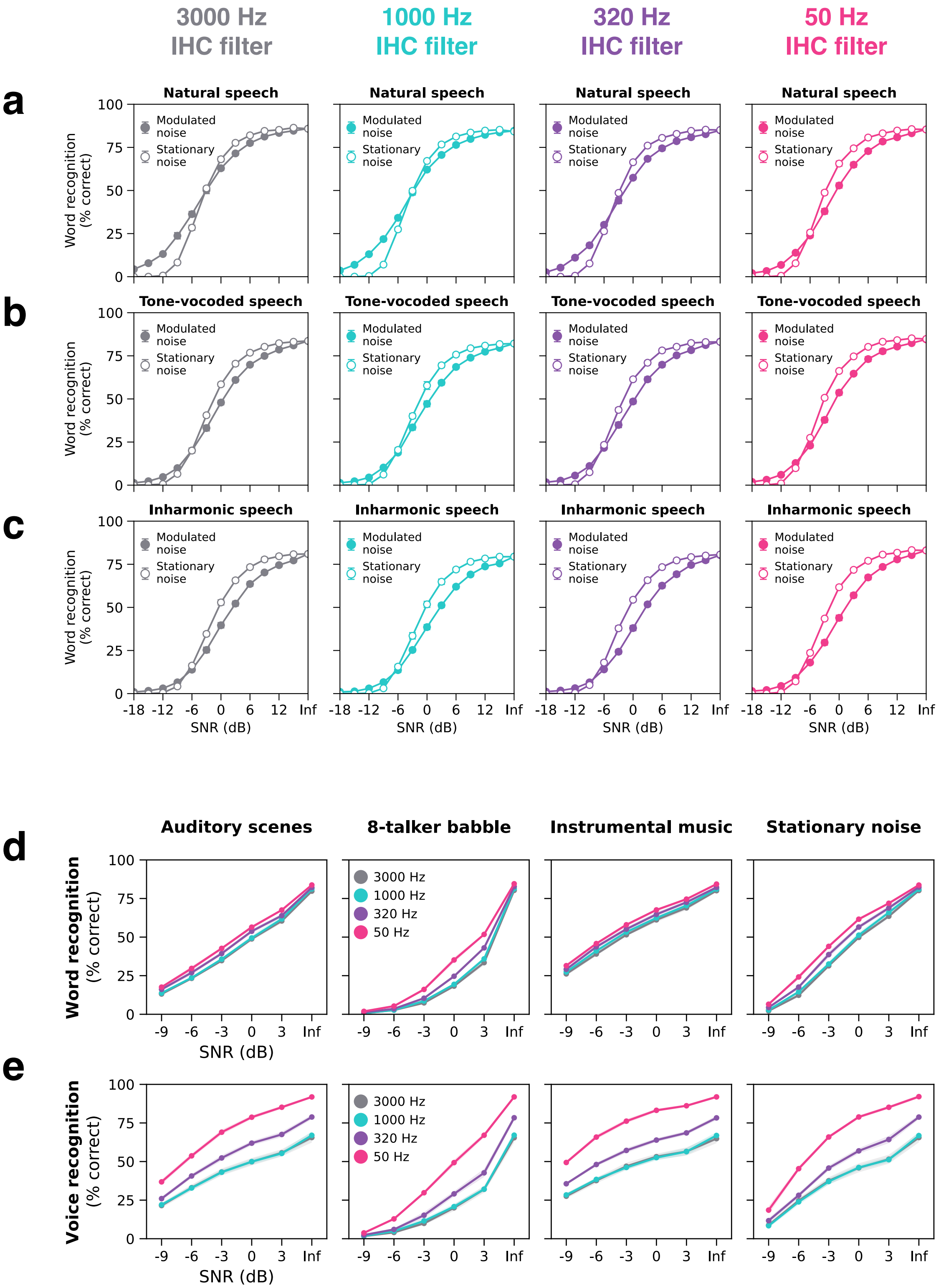

Supplementary Fig. 7 | Model word and voice recognition with inharmonic speech in noise. To directly compare effects of the inharmonicity and tone vocoding stimulus manipulations on model word recognition in noise, we measured word recognition accuracy in stationary and modulated speech-shaped noise at SNRs between -18 and +15 dB in 3 dB increments using (**a.**) natural, (**b.**) tone-vocoded, and (**c.**) inharmonic versions of the same speech. The tone-vocoded speech was fully vocoded (0 channels with intact TFS). **d.** Model word recognition with inharmonic speech as a function of SNR in four different types of real-world noise. **e.** Model voice recognition with inharmonic speech as a function of SNR in four different types of real-world noise. All error bars indicate ±2 standard errors of the mean across 10 network architectures.

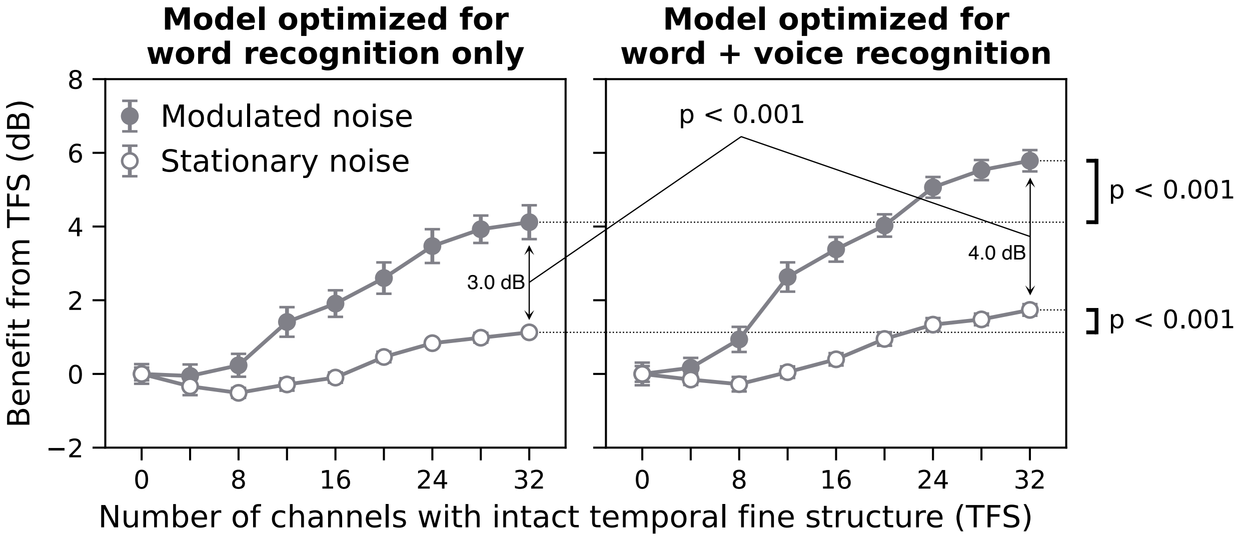

Supplementary Fig. 8 | Models optimized jointly for word and voice recognition exhibit a larger effect of tone vocoding than models optimized solely for word recognition. Tone vocoding results for 3000 Hz phase locking models optimized for either word recognition only (left panel) or word and voice recognition jointly (right panel). Plotting conventions are identical to Fig. 7c. Speech reception thresholds were measured using progressively tone-vocoded speech in noise. The benefit from temporal fine structure was quantified as the dB improvement in speech reception thresholds relative to performance with fully tone-vocoded speech (0 channels intact). The benefit from temporal fine structure is plotted as a function of the number of channels with intact temporal fine structure. Open circles plot the benefit in stationary noise and closed circles plot the benefit in amplitude-modulated noise. Error bars indicate ±2 standard errors of the mean across 10 network architectures. The statistical significance of differences between the two models was assessed by two-tailed paired comparisons against bootstrapped null distributions from the model optimized solely for word recognition. Exact p-values were 2.1e-14 (effect on stationary noise), and 4.4e-18 (effect on modulated noise), and 7.5e-10 (effect on difference between stationary and modulated noise).

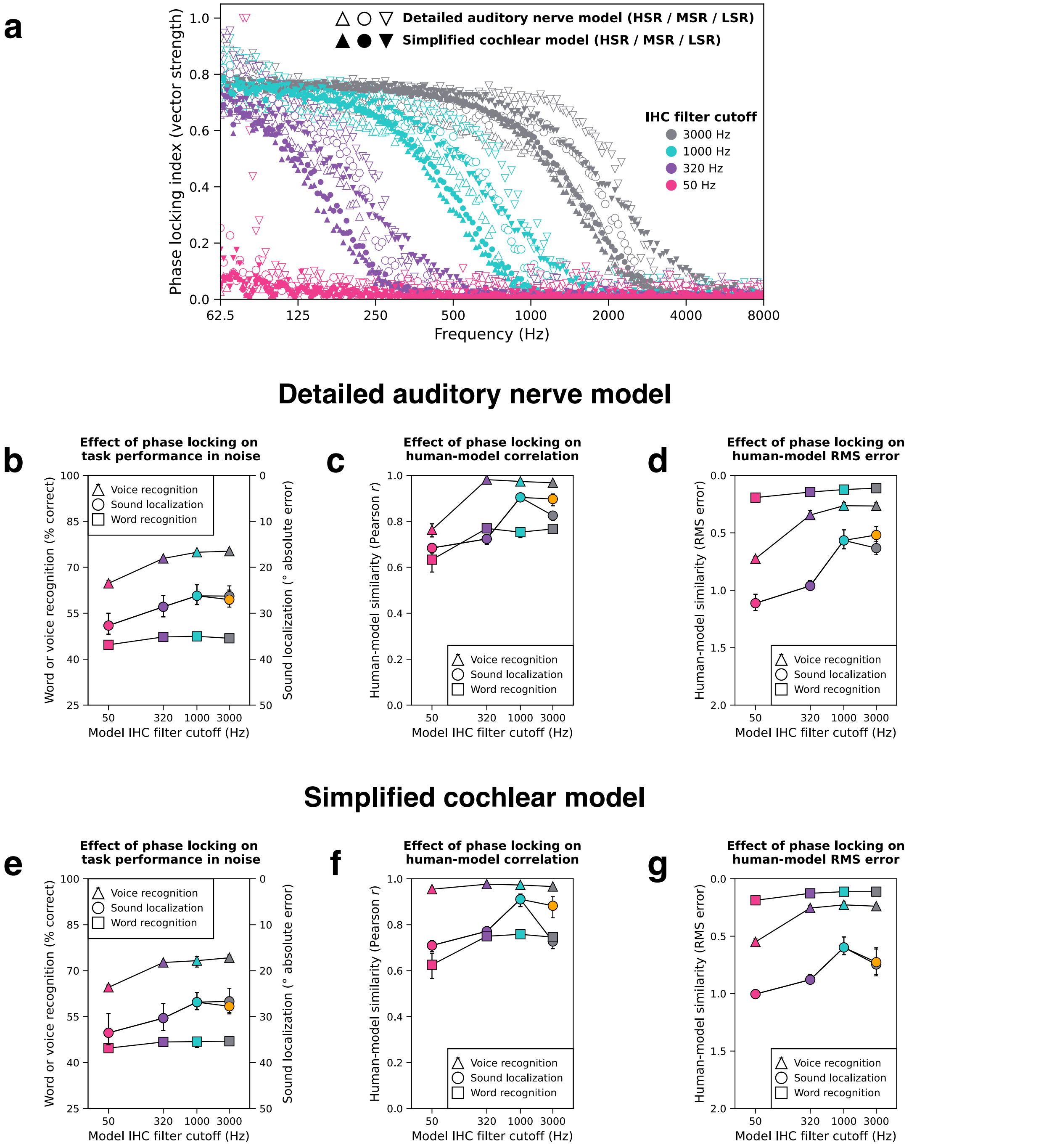

Supplementary Fig. 9 | Comparison of model results with detailed vs. simplified cochlear stages. a. The strength of phase locking as a function of frequency for simulated auditory nerve fibers under four inner hair cell (IHC) low-pass filter cutoffs (different colors). Nerve fibers simulated with the detailed auditory nerve model[5] (open symbols) and the simplified cochlear model (solid symbols) exhibit similar roll-offs in phase locking. The three different symbol shapes indicate high-, medium-, and low-spontaneous-rate (HSR, MSR, and LSR) auditory nerve fibers. Panels **b**, **c**, and **d** present results for models operating on input from the detailed auditory nerve model. **b.** Aggregate measures of task performance in noise as a function of phase locking. Word and voice recognition performance are plotted on the left y-axis (solid lines). Localization model performance is plotted on the right y-axis (dotted lines). **c.** Aggregate measure of human-model similarity (quantified as the Pearson correlation coefficient averaged across all experiments for each task) as a function of phase locking. **d.** Aggregate measure of human-model similarity (quantified as the min-max normalized root-mean-squared error averaged across all experiments for each task) as a function of phase locking. Panels **e**, **f**, and **g** are formatted identically to **b**, **c**, and **d** but present results for models operating on input from the simplified cochlear model. The orange symbol in panels **b** - **g** represents the 3000 Hz sound localization model with delayed interaural integration (see Fig. 4). Error bars indicate 95% confidence intervals bootstrapped across 10 network architectures for each model. We note that the model with the simplified cochlea stage exhibited thresholds for the pure tone lateralization experiment that were poor overall (see Supplementary Fig. 10d). This has a large impact on the RMS metric (accounting for the apparent lack of benefit of delayed binaural integration) even though the results are qualitatively similar to those of the model with the detailed peripheral stage (as is captured by the correlation metric).

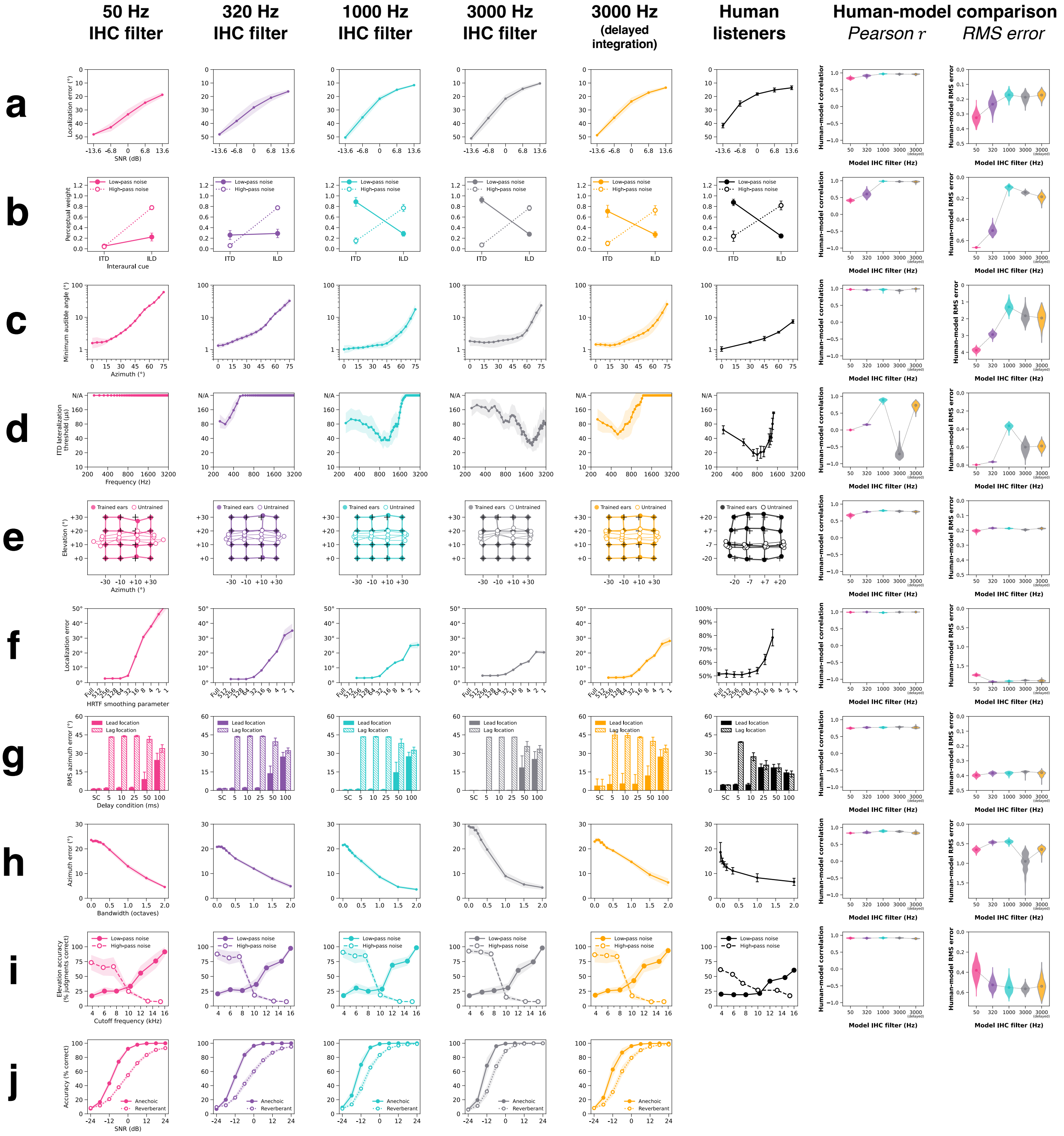

Supplementary Fig. 10 | Simplified cochlear model -- effect of phase locking on all sound localization experiments. This grid summarizes the behavioral data used to measure human-model similarity scores for localization models with the simplified cochlear stage (see Supplemental Fig. 2 for analogous results with detailed the auditory nerve model). The first four columns correspond to models optimized with different phase locking limits. The fifth (orange) column corresponds to the 3000 Hz phase locking model with network architectures modified to delay binaural integration. The sixth column contains results from human listeners. The rightmost two columns quantify human-similarity by measuring Pearson correlations and root-mean-squared error between analogous human and model data points. Violin plots depict bootstrapped distributions of human-model similarity scores across 10 network architectures per phase locking condition. Rows correspond to 10 different sound localization experiments. **a.** Sound localization in noise. **b.** Minimum audible angle vs. frequency. **c.** ITD / ILD cue weighting. **d.** ITD lateralization vs. frequency. **e.** Effect of changing ears. **f.** Effect of smoothing spectral cues. **g.** Precedence effect. **h.** Bandwidth dependency of localization. **i.** Median plane spectral cues. **j.** Speech localization in noise and reverberation (model experiment only). All model error bars indicate ±2 standard errors of the mean across 10 network architectures.

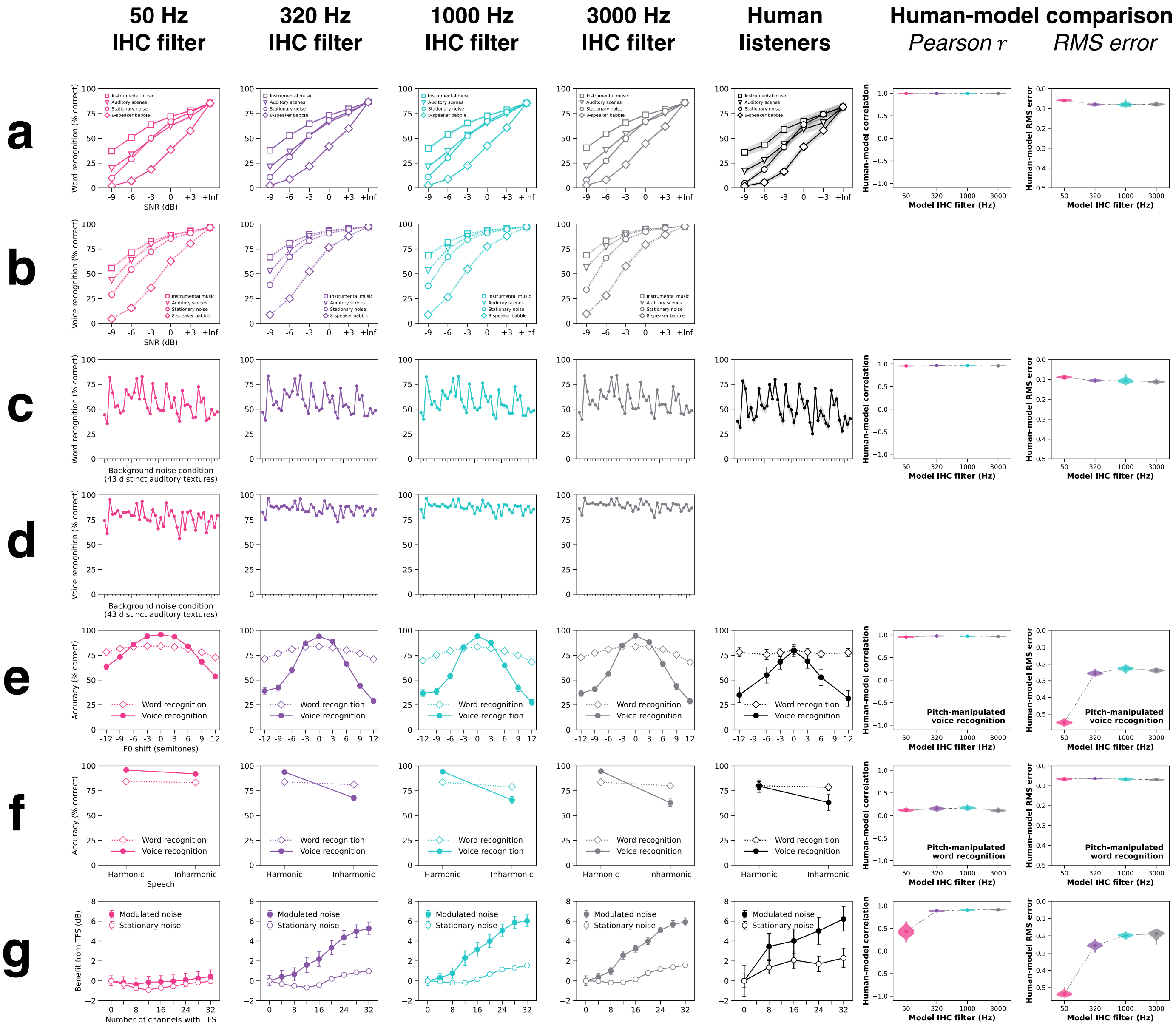

Supplementary Fig. 11 | Simplified cochlear model -- effect of phase locking on all speech experiments. This grid summarizes the behavioral data used to measure human-model similarity scores for the word and voice recognition models with the simplified cochlear stage (see Supplemental Fig. 5 for analogous results with the detailed auditory nerve model). The first four columns correspond to models optimized with different phase locking limits. The fifth column contains results from human listeners. The rightmost two columns quantify human-similarity by measuring Pearson correlations and root-mean-squared error between analogous human and model data points. Violin plots depict bootstrapped distributions of human-model similarity scores across 10 network architectures per phase locking condition. Rows correspond to 7 speech experiments. **a.** Word recognition in real-world noise conditions. **b.** Voice recognition in real-world noise conditions (model experiment only). **c.** Word recognition in 43 distinct auditory textures at -3 dB SNR. **d.** Voice recognition in 43 distinct auditory textures at -3 dB SNR (model experiment only). **e.** Word and voice recognition with F0-shifted speech. **f.** Word and voice recognition with harmonic and inharmonic speech. **g.** Effect of tone vocoding on word recognition in stationary and modulated noise. All model error bars indicate ±2 standard errors of the mean across 10 network architectures.

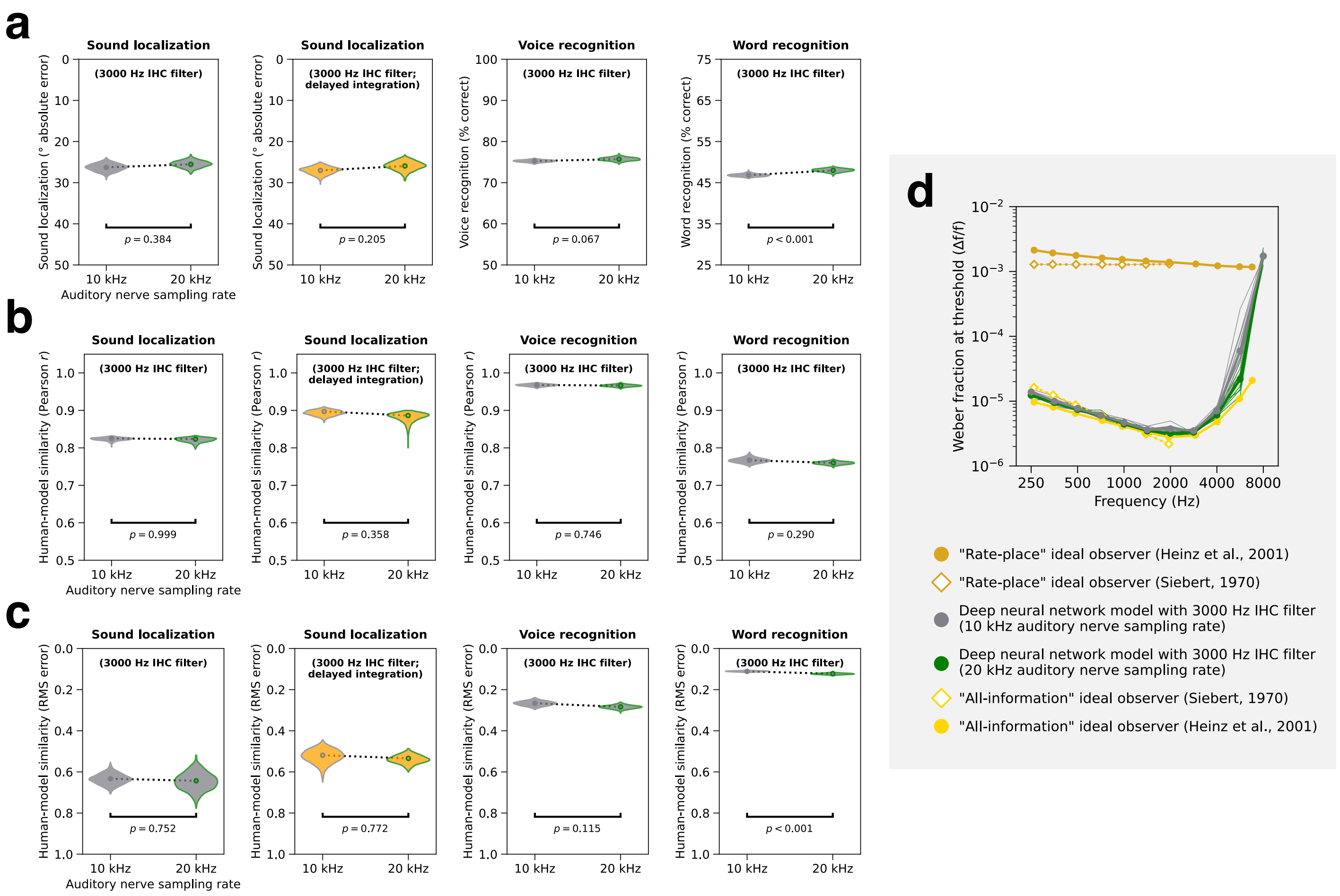

Supplementary Fig. 12 | Effect of increasing auditory nerve sampling rate from 10 to 20 kHz. (**a-c**) Each panel in this grid compares a measure of overall task performance (row **a**) or human-model similarity (rows **b** and **c**) between otherwise identical models with 10 or 20 kHz auditory nerve sampling rates. The four columns respectively feature results from sound localization models without delayed interaural integration, sound localization models with delayed interaural integration, voice recognition models, and word recognition models. All models had phase locking up to 3000 Hz. **a.** Effects on overall task performance in noise, quantified as mean absolute error for sound localization and percent correct for voice and word recognition. **b.** Effects on overall human-model similarity, quantified as the Pearson correlation coefficient averaged across all experiments for each task. **c.** Effects on overall human-model similarity, quantified as the root-mean-squared error min-max normalized and averaged across all experiments for each task. All y-axes are oriented such that higher positions correspond to better or more human-like task performance. Violin plots depict bootstrapped distributions across 10 network architectures. Two-tailed p-values indicate the probability of obtaining a score more extreme than the mean of the 20 kHz model under a bootstrapped null distribution from the 10 kHz model (p-values were not corrected for multiple comparisons). Results from the individual experiments are shown in Supplementary Fig. 13 (sound localization) and 14 (word and voice recognition). Overall, results were very similar for the two auditory nerve sampling rates. The two instances where there were statistically significant differences (word recognition task performance: p=2e-4; word recognition human-model RMS error: p=1.8e-6) were small in absolute terms. **d.** Effect of auditory nerve sampling rate on deep neural network frequency discrimination thresholds. Thresholds for the four ideal observer models (gold and yellow lines) and the 20 kHz sampling rate deep neural network model (green) are re-plotted from Fig. 8c. Here, we have added a 10 kHz sampling rate model (grey), which closely matches the 20 kHz model as well as the ideal observer "all-information" ideal observers. Deep neural network model thresholds are plotted as the mean across 10 network architectures for each phase locking conditions (thick green and grey lines; error bars indicate ±2 standard errors of the mean). Thin lines plot thresholds from individual network architectures.

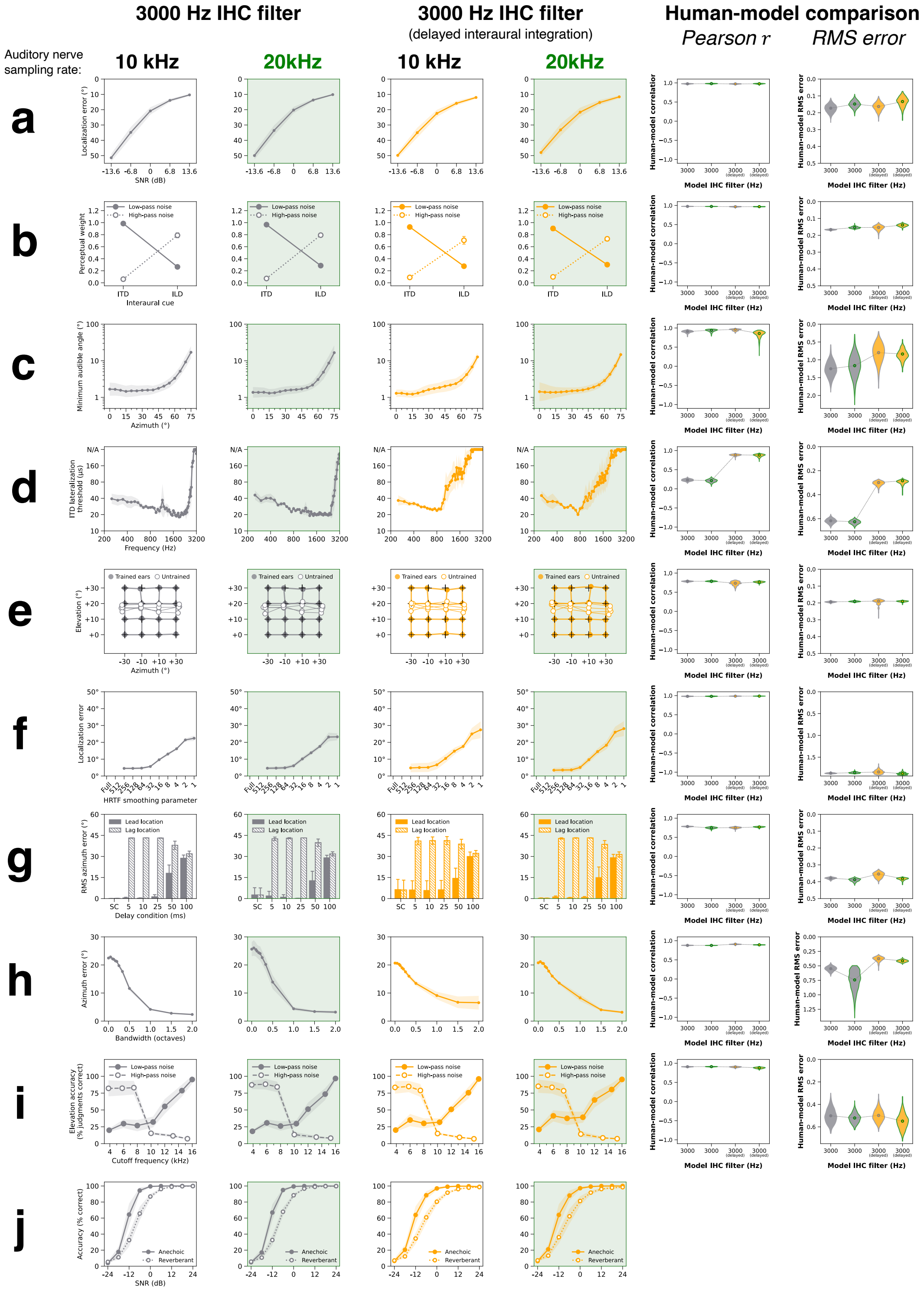

Supplementary Fig. 13 | Effect of increasing auditory nerve sampling rate from 10 to 20 kHz on all sound localization experiments. This grid compares results from sound localization models with auditory nerve sampling rates of either 10 kHz (first and third columns) or 20 kHz (second and fourth columns; highlighted green). All models had phase locking up to 3000 Hz. The first and second columns contain results from models without delayed interaural integration. The third and fourth columns contain results from models with delayed interaural integration. The fifth and sixth columns quantify human-similarity by measuring Pearson correlations and root-mean-squared error between analogous human and model data points. Violin plots depict bootstrapped distributions of human-model similarity scores across 10 network architectures per condition. Rows correspond to 10 different sound localization experiments. **a.** Sound localization in noise. **b.** Minimum audible angle vs. frequency. **c.** ITD / ILD cue weighting. **d.** ITD lateralization vs. frequency. **e.** Effect of changing ears. **f.** Effect of smoothing spectral cues. **g.** Precedence effect. **h.** Bandwidth dependency of localization. **i.** Median plane spectral cues. **j.** Speech localization in noise and reverberation (model experiment only). All model error bars indicate ±2 standard errors of the mean across 10 network architectures. Overall, results were very similar for the two auditory nerve sampling rates.

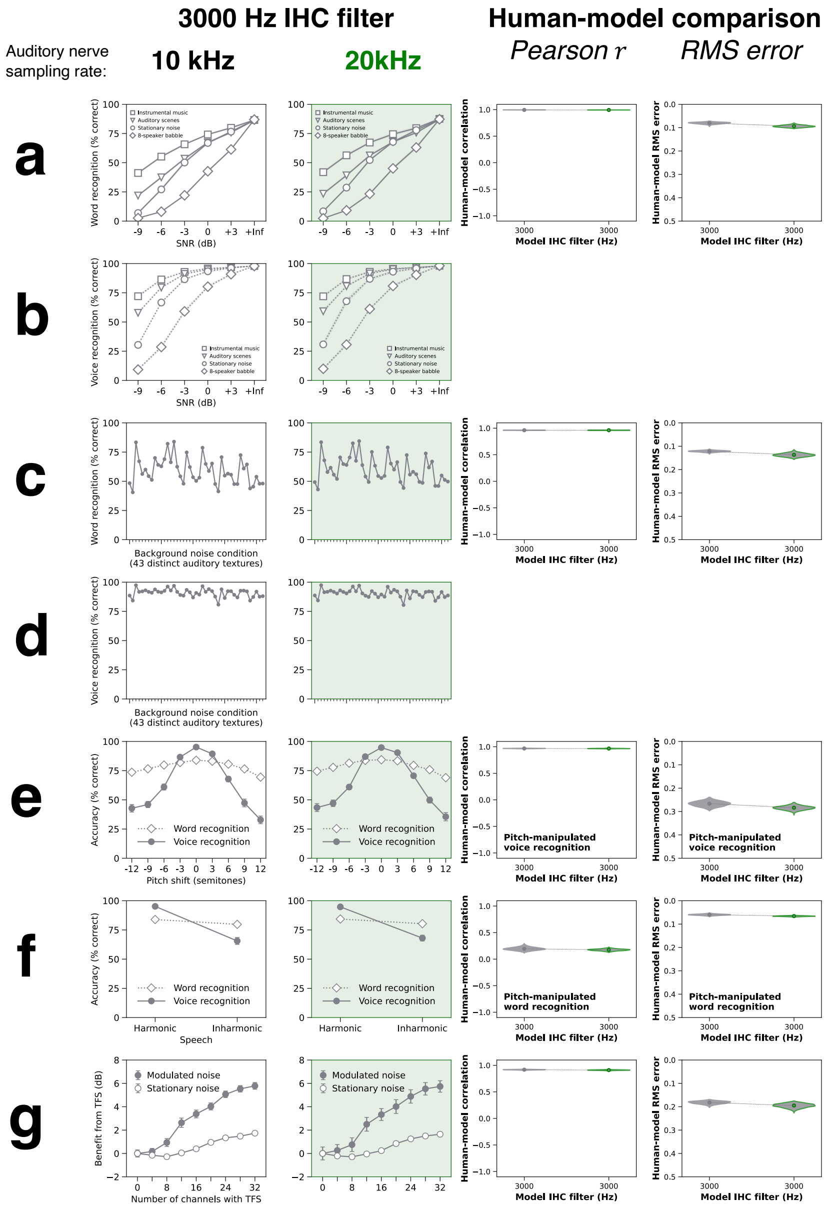

Supplementary Fig. 14 | Effect of increasing auditory nerve sampling rate from 10 to 20 kHz on all speech experiments. This grid compares results from word and voice recognition models with auditory nerve sampling rates of either 10 kHz (first column) or 20 kHz (second column; highlighted green). The third and fourth columns quantify human-similarity by measuring Pearson correlations and root-mean-squared error between analogous human and model data points. Violin plots depict bootstrapped distributions of human-model similarity scores across 10 network architectures per condition. Rows correspond to 7 speech experiments. **a.** Word recognition in real-world noise conditions. **b.** Voice recognition in real-world noise conditions (model experiment only). **c.** Word recognition in 43 distinct auditory textures at -3 dB SNR. **d.** Voice recognition in 43 distinct auditory textures at -3 dB SNR (model experiment only). **e.** Word and voice recognition with F0-shifted speech. **f.** Word and voice recognition with harmonic and inharmonic speech. **g.** Effect of tone vocoding on word recognition in stationary and modulated noise. All model error bars indicate ±2 standard errors of the mean across 10 network architectures. Overall, results were very similar for the two auditory nerve sampling rates.

Architecture	arch_01	arch_02	arch_03	arch_04	arch_05	arch_06	arch_07	arch_08	arch_09	arch_10
Operation	input [50,10000,6]	input [50,10000,6]	input [50,10000,6]	input [50,10000,6]	input [50,10000,6]	input [50,10000,6]	input [50,10000,6]	input [50,10000,6]	input [50,10000,6]	input [50,10000,6]
1	conv0 [1, 8, 32]	conv0 [2, 8, 32]	conv0 [1, 4, 32]	conv0 [3, 8, 32]	conv0 [2, 32, 32]	conv0 [1, 64, 32]	conv0 [1, 16, 32]	conv0 [1, 64, 32]	conv0 [3, 32, 32]	conv0 [2, 4, 32]
2	mpool0 [1, 1]	mpool0 [1, 1]	mpool0 [1, 1]	mpool0 [1, 1]	mpool0 [1, 2]	mpool0 [1, 8]	mpool0 [1, 1]	mpool0 [1, 1]	mpool0 [1, 1]	mpool0 [2, 2]
3	relu0	relu0	relu0	relu0	relu0	relu0	relu0	relu0	relu0	relu0
4	bnorm0	bnorm0	bnorm0	bnorm0	bnorm0	bnorm0	bnorm0	bnorm0	bnorm0	bnorm0
5	conv1 [1, 64, 32]	conv1 [3, 16, 32]	conv1 [3, 32, 32]	conv1 [3, 8, 32]	conv1 [1, 4, 64]	conv1 [2, 4, 64]	conv1 [1, 8, 32]	conv1 [2, 16, 32]	conv1 [2, 16, 32]	conv1 [2, 4, 32]
6	mpool1 [1, 1]	mpool1 [1, 1]	mpool1 [1, 8]	mpool1 [1, 2]	mpool1 [1, 4]	mpool1 [1, 1]	mpool1 [1, 2]	mpool1 [1, 8]	mpool1 [1, 4]	mpool1 [1, 4]
7	relu1	relu1	relu1	relu1	relu1	relu1	relu1	relu1	relu1	relu1
8	bnorm1	bnorm1	bnorm1	bnorm1	bnorm1	bnorm1	bnorm1	bnorm1	bnorm1	bnorm1
9	conv2 [1, 64, 32]	conv2 [2, 4, 32]	conv2 [3, 32, 64]	conv2 [1, 32, 64]	conv2 [3, 2, 64]	conv2 [1, 32, 64]	conv2 [2, 4, 64]	conv2 [2, 4, 64]	conv2 [2, 32, 64]	conv2 [3, 16, 64]
10	mpool2 [1, 8]	mpool2 [1, 8]	mpool2 [1, 1]	mpool2 [1, 1]	mpool2 [1, 1]	mpool2 [2, 4]	mpool2 [1, 1]	mpool2 [1, 1]	mpool2 [1, 1]	mpool2 [1, 2]
11	relu2	relu2	relu2	relu2	relu2	relu2	relu2	relu2	relu2	relu2
12	bnorm2	bnorm2	bnorm2	bnorm2	bnorm2	bnorm2	bnorm2	bnorm2	bnorm2	bnorm2
13	conv3 [2, 4, 64]	conv3 [3, 16, 64]	conv3 [1, 8, 64]	conv3 [3, 8, 64]	conv3 [2, 8, 64]	conv3 [3, 4, 128]	conv3 [2, 32, 64]	conv3 [2, 16, 64]	conv3 [3, 4, 64]	conv3 [1, 2, 128]
14	mpool3 [2, 4]	mpool3 [1, 1]	mpool3 [1, 4]	mpool3 [2, 4]	mpool3 [1, 1]	mpool3 [1, 1]	mpool3 [1, 4]	mpool3 [1, 1]	mpool3 [1, 4]	mpool3 [1, 2]
15	relu3	relu3	relu3	relu3	relu3	relu3	relu3	relu3	relu3	relu3
16	bnorm3	bnorm3	bnorm3	bnorm3	bnorm3	bnorm3	bnorm3	bnorm3	bnorm3	bnorm3
17	conv4 [3, 8, 128]	conv4 [1, 8, 64]	conv4 [3, 8, 64]	conv4 [2, 2, 128]	conv4 [1, 16, 64]	conv4 [2, 16, 128]	conv4 [3, 2, 64]	conv4 [1, 16, 64]	conv4 [3, 8, 128]	flatten
18	mpool4 [1, 1]	mpool4 [1, 4]	mpool4 [1, 1]	mpool4 [1, 4]	mpool4 [1, 4]	mpool4 [1, 2]	mpool4 [1, 1]	mpool4 [1, 2]	mpool4 [1, 4]	fc0 [512]
19	relu4	relu4	relu4	relu4	relu4	relu4	relu4	relu4	relu4	relu_fc0
20	bnorm4	bnorm4	bnorm4	bnorm4	bnorm4	bnorm4	bnorm4	bnorm4	bnorm4	bnorm_fc0
21	conv5 [3, 32, 128]	conv5 [3, 8, 128]	conv5 [1, 2, 64]	conv5 [1, 4, 256]	conv5 [3, 4, 128]	conv5 [1, 2, 256]	conv5 [1, 2, 64]	conv5 [2, 32, 128]	conv5 [3, 2, 256]	dropout
22	mpool5 [1, 4]	mpool5 [1, 4]	mpool5 [1, 1]	mpool5 [1, 1]	mpool5 [1, 2]	mpool5 [1, 1]	mpool5 [2, 4]	mpool5 [1, 4]	mpool5 [1, 2]	fc [504]
23	relu5	relu5	relu5	relu5	relu5	relu5	relu5	relu5	relu5	
24	bnorm5	bnorm5	bnorm5	bnorm5	bnorm5	bnorm5	bnorm5	bnorm5	bnorm5	
25	conv6 [3, 4, 256]	conv6 [2, 2, 128]	conv6 [2, 2, 64]	conv6 [3, 2, 256]	conv6 [3, 4, 256]	conv6 [3, 4, 256]	conv6 [1, 8, 128]	conv6 [2, 16, 128]	conv6 [2, 8, 512]	
26	mpool6 [1, 1]	mpool6 [1, 2]	mpool6 [2, 4]	mpool6 [1, 1]	mpool6 [1, 1]	mpool6 [1, 2]	mpool6 [1, 1]	mpool6 [1, 1]	mpool6 [1, 1]	
27	relu6	relu6	relu6	relu6	relu6	relu6	relu6	relu6	relu6	
28	bnorm6	bnorm6	bnorm6	bnorm6	bnorm6	bnorm6	bnorm6	bnorm6	bnorm6	
29	conv7 [3, 8, 256]	conv7 [3, 2, 256]	conv7 [2, 4, 128]	conv7 [2, 2, 256]	conv7 [3, 4, 256]	flatten	flatten	conv7 [1, 2, 128]	conv7 [3, 4, 512]	
30	mpool7 [1, 2]	mpool7 [1, 2]	mpool7 [1, 1]	mpool7 [1, 2]	mpool7 [1, 1]	fc0 [512]	fc0 [512]	mpool7 [1, 1]	mpool7 [1, 2]	
31	relu7	relu7	relu7	relu7	relu7	relu_fc0	relu_fc0	relu7	relu7	
32	bnorm7	bnorm7	bnorm7	bnorm7	bnorm7	bnorm_fc0	bnorm_fc0	bnorm7	bnorm7	
33	flatten	conv8 [1, 8, 512]	conv8 [1, 8, 128]	flatten	conv8 [2, 4, 256]	dropout	dropout	conv8 [3, 16, 128]	conv8 [1, 3, 512]	
34	fc0 [512]	mpool8 [1, 2]	mpool8 [1, 1]	fc0 [512]	mpool8 [1, 2]	fc [504]	fc [504]	mpool8 [1, 4]	mpool8 [1, 1]	
35	relu_fc0	relu8	relu8	relu_fc0	relu8			relu8	relu8	
36	bnorm_fc0	bnorm8	bnorm8	bnorm_fc0	bnorm8			bnorm8	bnorm8	
37	dropout	flatten	conv9 [3, 2, 128]	dropout	flatten			flatten	flatten	
38	fc [504]	fc0 [512]	mpool9 [1, 4]	fc [504]	fc0 [512]			fc0 [512]	fc0 [512]	
39		relu_fc0	relu9		relu_fc0			relu_fc0	relu_fc0	
40		bnorm_fc0	bnorm9		bnorm_fc0			bnorm_fc0	bnorm_fc0	
41		dropout	flatten		dropout			dropout	dropout	
42		fc [504]	fc0 [512]		fc [504]			fc [504]	fc [504]	
43			relu_fc0							
44			bnorm_fc0							
45			dropout							
46			fc [504]							
47										

Supplementary Table 1 | Neural network architectures for sound localization models. Grey bands indicate blocks of convolution, pooling, nonlinear rectification, and normalization operations. The convolution operations highlighted in orange were replaced with grouped convolutions (2 groups for the left and right ear) when network architectures were modified to delay binaural integration. Legend:

- $conv\ [h, w, k]$: convolutional layer with h = kernel height (frequency dimension), w = kernel width (time dimension), and k = number of kernels
- $relu$: rectified linear unit activation function
- $mpool\ [s_f, s_t]$: max pooling operation with stride s_f in the frequency dimension and stride s_t in the time dimension
- $bnorm$: batch normalization operation
- $flatten$: multidimensional representation reshaped to a vector
- $fc\ [N]$: fully-connected layer with N units
- $dropout$: dropout regularization with 50% dropout rate

Architecture	arch0_0000	arch0_0001	arch0_0002	arch0_0004	arch0_0006	arch0_0007	arch0_0008	arch0_0009	arch0_0016	arch0_0017
Operation	input [50, 20000, 3]	input [50, 20000, 3]	input [50, 20000, 3]	input [50, 20000, 3]	input [50, 20000, 3]	input [50, 20000, 3]	input [50, 20000, 3]	input [50, 20000, 3]	input [50, 20000, 3]	input [50, 20000, 3]
1	input_lnorm	input_lnorm	input_lnorm	input_lnorm	input_lnorm	input_lnorm	input_lnorm	input_lnorm	input_lnorm	input_lnorm
2	conv0 [2, 42, 32]	conv0 [1, 84, 32]	conv0 [4, 21, 32]	conv0 [2, 42, 32]	conv0 [2, 42, 32]	conv0 [2, 42, 32]	conv0 [2, 42, 32]	conv0 [2, 42, 32]	conv0 [2, 42, 32]	conv0 [2, 42, 32]
3	relu0	relu0	relu0	relu0	relu0	relu0	relu0	relu0	relu0	relu0
4	hpool0 [2, 4]	hpool0 [2, 4]	hpool0 [2, 4]	hpool0 [2, 4]	hpool0 [2, 4]	hpool0 [2, 4]	hpool0 [2, 4]	hpool0 [2, 4]	hpool0 [2, 4]	hpool0 [2, 4]
5	lnorm0	lnorm0	lnorm0	lnorm0	lnorm0	lnorm0	lnorm0	lnorm0	lnorm0	lnorm0
6	conv1 [2, 18, 64]	conv1 [2, 18, 64]	conv1 [2, 18, 64]	conv1 [4, 9, 64]	conv1 [2, 18, 64]	conv1 [2, 18, 64]	conv1 [2, 18, 64]	conv1 [2, 18, 64]	conv1 [2, 18, 64]	conv1 [2, 18, 64]
7	relu1	relu1	relu1	relu1	relu1	relu1	relu1	relu1	relu1	relu1
8	hpool1 [2, 4]	hpool1 [2, 4]	hpool1 [2, 4]	hpool1 [2, 4]	hpool1 [2, 4]	hpool1 [2, 4]	hpool1 [2, 4]	hpool1 [2, 4]	hpool1 [2, 4]	hpool1 [2, 4]
9	lnorm1	lnorm1	lnorm1	lnorm1	lnorm1	lnorm1	lnorm1	lnorm1	lnorm1	lnorm1
10	conv2 [6, 6, 128]	conv2 [6, 6, 128]	conv2 [6, 6, 128]	conv2 [6, 6, 128]	conv2 [12, 3, 128]	conv2 [6, 6, 128]	conv2 [6, 6, 128]	conv2 [6, 6, 128]	conv2 [6, 6, 128]	conv2 [6, 6, 128]
11	relu2	relu2	relu2	relu2	relu2	relu2	relu2	relu2	relu2	relu2
12	hpool2 [1, 4]	hpool2 [1, 4]	hpool2 [1, 4]	hpool2 [1, 4]	hpool2 [1, 4]	hpool2 [1, 4]	hpool2 [1, 4]	hpool2 [1, 4]	hpool2 [1, 4]	hpool2 [1, 4]
13	lnorm2	lnorm2	lnorm2	lnorm2	lnorm2	lnorm2	lnorm2	lnorm2	lnorm2	lnorm2
14	conv3 [6, 6, 256]	conv3 [6, 6, 256]	conv3 [6, 6, 256]	conv3 [6, 6, 256]	conv3 [6, 6, 256]	conv3 [3, 12, 256]	conv3 [12, 3, 256]	conv3 [6, 6, 256]	conv3 [6, 6, 256]	conv3 [6, 6, 256]
15	relu3	relu3	relu3	relu3	relu3	relu3	relu3	relu3	relu3	relu3
16	hpool3 [1, 4]	hpool3 [1, 4]	hpool3 [1, 4]	hpool3 [1, 4]	hpool3 [1, 4]	hpool3 [1, 4]	hpool3 [1, 4]	hpool3 [1, 4]	hpool3 [1, 4]	hpool3 [1, 4]
17	lnorm3	lnorm3	lnorm3	lnorm3	lnorm3	lnorm3	lnorm3	lnorm3	lnorm3	lnorm3
18	conv4 [8, 8, 512]	conv4 [8, 8, 512]	conv4 [8, 8, 512]	conv4 [8, 8, 512]	conv4 [8, 8, 512]	conv4 [8, 8, 512]	conv4 [8, 8, 512]	conv4 [4, 16, 512]	conv4 [8, 8, 512]	conv4 [8, 8, 512]
19	relu4	relu4	relu4	relu4	relu4	relu4	relu4	relu4	relu4	relu4
20	hpool4 [1, 1]	hpool4 [1, 1]	hpool4 [1, 1]	hpool4 [1, 1]	hpool4 [1, 1]	hpool4 [1, 1]	hpool4 [1, 1]	hpool4 [1, 1]	hpool4 [1, 1]	hpool4 [1, 1]
21	lnorm4	lnorm4	lnorm4	lnorm4	lnorm4	lnorm4	lnorm4	lnorm4	lnorm4	lnorm4
22	conv5 [6, 6, 512]	conv5 [6, 6, 512]	conv5 [6, 6, 512]	conv5 [6, 6, 512]	conv5 [6, 6, 512]	conv5 [6, 6, 512]	conv5 [6, 6, 512]	conv5 [6, 6, 512]	conv5 [6, 6, 512]	conv5 [6, 6, 512]
23	relu5	relu5	relu5	relu5	relu5	relu5	relu5	relu5	relu5	relu5
24	hpool5 [1, 1]	hpool5 [1, 1]	hpool5 [1, 1]	hpool5 [1, 1]	hpool5 [1, 1]	hpool5 [1, 1]	hpool5 [1, 1]	hpool5 [1, 1]	hpool5 [1, 1]	hpool5 [1, 1]
25	lnorm5	lnorm5	lnorm5	lnorm5	lnorm5	lnorm5	lnorm5	lnorm5	lnorm5	lnorm5
26	conv6 [8, 8, 512]	conv6 [8, 8, 512]	conv6 [8, 8, 512]	conv6 [8, 8, 512]	conv6 [8, 8, 512]	conv6 [8, 8, 512]	conv6 [8, 8, 512]	conv6 [8, 8, 512]	conv6 [8, 8, 512]	conv6 [8, 8, 512]
27	relu6	relu6	relu6	relu6	relu6	relu6	relu6	relu6	relu6	relu6
28	hpool6 [2, 4]	hpool6 [2, 4]	hpool6 [2, 4]	hpool6 [2, 4]	hpool6 [2, 4]	hpool6 [2, 4]	hpool6 [2, 4]	hpool6 [2, 4]	hpool6 [2, 4]	hpool6 [2, 4]
29	lnorm6	lnorm6	lnorm6	lnorm6	lnorm6	lnorm6	lnorm6	lnorm6	lnorm6	lnorm6
30	flatten	flatten	flatten	flatten	flatten	flatten	flatten	flatten	conv7 [2, 8, 512]	conv7 [8, 2, 512]
31	fc0 [512]	fc0 [512]	fc0 [512]	fc0 [512]	fc0 [512]	fc0 [512]	fc0 [512]	fc0 [512]	relu7	relu7
32	relu_fc0	relu_fc0	relu_fc0	relu_fc0	relu_fc0	relu_fc0	relu_fc0	relu_fc0	hpool7 [1, 1]	hpool7 [1, 1]
33	lnorm_fc0	lnorm_fc0	lnorm_fc0	lnorm_fc0	lnorm_fc0	lnorm_fc0	lnorm_fc0	lnorm_fc0	lnorm7	lnorm7
34	dropout	dropout	dropout	dropout	dropout	dropout	dropout	dropout	flatten	flatten
35	fc [433, 794]	fc [433, 794]	fc [433, 794]	fc [433, 794]	fc [433, 794]	fc [433, 794]	fc [433, 794]	fc [433, 794]	fc0 [512]	fc0 [512]
36									relu_fc0	relu_fc0
37									lnorm_fc0	lnorm_fc0
38									dropout	dropout
39									fc [433, 794]	fc [433, 794]
40										

Supplementary Table 2 | Neural network architectures for word and voice recognition models. Grey bands indicate blocks of convolution, pooling, nonlinear rectification, and normalization operations. For networks jointly optimized for word and voice recognition, there were two fully-connected read-out layers in parallel, one for each task (433 units for voice recognition and 794 units for word recognition). Legend:

- $conv\ [h, w, k]$: convolutional layer with $h =$ kernel height (frequency dimension), $w =$ kernel width (time dimension), and $k =$ number of kernels
- $relu$: rectified linear unit activation function
- $hpool\ [s_f, s_t]$: Hanning window weighted averaged pooling operation with stride s_f in the frequency dimension and stride s_t in the time dimension
- $lnorm$: layer normalization operation
- $flatten$: multidimensional representation reshaped to a vector
- $fc\ [N]$: fully-connected layer with N units
- $fc\ [N_{voice}, N_{word}]$: two parallel fully-connected layers operating on the same input, one with N_{voice} units and one with N_{word} units
- $dropout$: dropout regularization with 50% dropout rate

Architecture	arch_f00	arch_f04	arch_f05	arch_f06	arch_f09	arch_f11	arch_f13	arch_f15	arch_f17	arch_f20
**Operation**	input [60, 5000, 2]	input [60, 5000, 2]	input [60, 5000, 2]	input [60, 5000, 2]	input [60, 5000, 2]	input [60, 5000, 2]	input [60, 5000, 2]	input [60, 5000, 2]	input [60, 5000, 2]	input [60, 5000, 2]
1	conv0 [3, 53, 32]	conv0 [3, 53, 64]	conv0 [3, 53, 32]	conv0 [3, 53, 64]	conv0 [3, 53, 64]	conv0 [3, 53, 32]	conv0 [3, 53, 32]	conv0 [3, 53, 64]	conv0 [3, 53, 64]	conv0 [3, 53, 64]
2	relu0	relu0	relu0	relu0	relu0	relu0	relu0	relu0	relu0	relu0
3	hpool0 [1, 2]	hpool0 [1, 2]	hpool0 [1, 2]	hpool0 [1, 2]	hpool0 [1, 2]	hpool0 [1, 2]	hpool0 [1, 2]	hpool0 [1, 2]	hpool0 [1, 2]	hpool0 [1, 2]
4	bnorm0	bnorm0	bnorm0	bnorm0	bnorm0	bnorm0	bnorm0	bnorm0	bnorm0	bnorm0
5	conv1 [1, 60, 64]	conv1 [1, 60, 64]	conv1 [1, 60, 128]	conv1 [1, 60, 128]	conv1 [3, 20, 128]	conv1 [1, 60, 64]	conv1 [3, 20, 64]	conv1 [1, 60, 64]	conv1 [1, 60, 128]	conv1 [3, 20, 128]
6	relu1	relu1	relu1	relu1	relu1	relu1	relu1	relu1	relu1	relu1
7	hpool1 [2, 4]	hpool1 [2, 4]	hpool1 [2, 4]	hpool1 [2, 4]	hpool1 [2, 4]	hpool1 [2, 4]	hpool1 [2, 4]	hpool1 [2, 4]	hpool1 [2, 4]	hpool1 [2, 4]
8	bnorm1	bnorm1	bnorm1	bnorm1	bnorm1	bnorm1	bnorm1	bnorm1	bnorm1	bnorm1
9	conv2 [3, 46, 128]	conv2 [3, 46, 128]	conv2 [3, 46, 128]	conv2 [3, 46, 128]	conv2 [3, 46, 128]	conv2 [3, 46, 128]	conv2 [3, 46, 128]	conv2 [3, 46, 128]	conv2 [3, 46, 128]	conv2 [3, 46, 128]
10	relu2	relu2	relu2	relu2	relu2	relu2	relu2	relu2	relu2	relu2
11	hpool2 [1, 6]	hpool2 [1, 6]	hpool2 [1, 6]	hpool2 [1, 6]	hpool2 [1, 6]	hpool2 [1, 6]	hpool2 [1, 6]	hpool2 [1, 6]	hpool2 [1, 6]	hpool2 [1, 6]
12	bnorm2	bnorm2	bnorm2	bnorm2	bnorm2	bnorm2	bnorm2	bnorm2	bnorm2	bnorm2
13	conv3 [8, 1, 256]	conv3 [8, 1, 256]	conv3 [8, 1, 256]	conv3 [8, 1, 256]	conv3 [8, 1, 256]	conv3 [8, 1, 256]	conv3 [8, 1, 256]	conv3 [8, 1, 256]	conv3 [8, 1, 256]	conv3 [8, 1, 256]
14	relu3	relu3	relu3	relu3	relu3	relu3	relu3	relu3	relu3	relu3
15	hpool3 [2, 2]	hpool3 [2, 2]	hpool3 [2, 2]	hpool3 [2, 2]	hpool3 [2, 2]	hpool3 [2, 2]	hpool3 [2, 2]	hpool3 [2, 2]	hpool3 [2, 2]	hpool3 [2, 2]
16	bnorm3	bnorm3	bnorm3	bnorm3	bnorm3	bnorm3	bnorm3	bnorm3	bnorm3	bnorm3
17	conv4 [7, 2, 256]	conv4 [7, 2, 256]	conv4 [7, 2, 256]	conv4 [7, 2, 256]	conv4 [7, 2, 256]	conv4 [7, 2, 256]	conv4 [7, 2, 256]	conv4 [7, 2, 256]	conv4 [7, 2, 256]	conv4 [7, 2, 256]
18	relu4	relu4	relu4	relu4	relu4	relu4	relu4	relu4	relu4	relu4
19	hpool4 [1, 1]	hpool4 [1, 1]	hpool4 [1, 1]	hpool4 [1, 1]	hpool4 [1, 1]	hpool4 [1, 1]	hpool4 [1, 1]	hpool4 [1, 1]	hpool4 [1, 1]	hpool4 [1, 1]
20	bnorm4	bnorm4	bnorm4	bnorm4	bnorm4	bnorm4	bnorm4	bnorm4	bnorm4	bnorm4
21	conv5 [2, 2, 512]	conv5 [2, 2, 512]	conv5 [2, 2, 512]	conv5 [2, 2, 512]	conv5 [2, 2, 512]	conv5 [2, 2, 512]	conv5 [2, 2, 512]	conv5 [2, 2, 512]	conv5 [2, 2, 512]	conv5 [2, 2, 512]
22	relu5	relu5	relu5	relu5	relu5	relu5	relu5	relu5	relu5	relu5
23	hpool5 [2, 1]	hpool5 [2, 1]	hpool5 [2, 1]	hpool5 [2, 1]	hpool5 [2, 1]	hpool5 [2, 1]	hpool5 [2, 1]	hpool5 [2, 1]	hpool5 [2, 1]	hpool5 [2, 1]
24	bnorm5	bnorm5	bnorm5	bnorm5	bnorm5	bnorm5	bnorm5	bnorm5	bnorm5	bnorm5
25	conv6 [1, 1, 512]	conv6 [1, 1, 512]	conv6 [1, 1, 512]	conv6 [1, 1, 512]	conv6 [1, 1, 512]	conv6 [1, 1, 512]	conv6 [1, 1, 512]	conv6 [1, 1, 512]	conv6 [1, 1, 512]	conv6 [1, 1, 512]
26	relu6	relu6	relu6	relu6	relu6	relu6	relu6	relu6	relu6	relu6
27	hpool6 [1, 1]	hpool6 [1, 1]	hpool6 [1, 1]	hpool6 [1, 1]	hpool6 [1, 1]	hpool6 [1, 1]	hpool6 [1, 1]	hpool6 [1, 1]	hpool6 [1, 1]	hpool6 [1, 1]
28	bnorm6	bnorm6	bnorm6	bnorm6	bnorm6	bnorm6	bnorm6	bnorm6	bnorm6	bnorm6
29	flatten	flatten	flatten	flatten	flatten	flatten	flatten	flatten	flatten	flatten
30	fc0 [512]	fc0 [512]	fc0 [512]	fc0 [512]	fc0 [512]	fc0 [1024]	fc0 [1024]	fc0 [1024]	fc0 [1024]	fc0 [1024]
31	relu_fc0	relu_fc0	relu_fc0	relu_fc0	relu_fc0	relu_fc0	relu_fc0	relu_fc0	relu_fc0	relu_fc0
32	bnorm_fc0	bnorm_fc0	bnorm_fc0	bnorm_fc0	bnorm_fc0	bnorm_fc0	bnorm_fc0	bnorm_fc0	bnorm_fc0	bnorm_fc0
33	dropout	dropout	dropout	dropout	dropout	dropout	dropout	dropout	dropout	dropout
34	fc [1]	fc [1]	fc [1]	fc [1]	fc [1]	fc [1]	fc [1]	fc [1]	fc [1]	fc [1]
35										

Supplementary Table 3 | Neural network architectures for frequency discrimination models. Grey bands indicate blocks of convolution, pooling, nonlinear rectification, and normalization operations. Networks operated on auditory nerve representations of two pure tones with different frequencies and were tasked with reporting which tone had a higher frequency (binary classification). Legend:

- $conv\ [h, w, k]$: convolutional layer with $h =$ kernel height (frequency dimension), $w =$ kernel width (time dimension), and $k =$ number of kernels
- $relu$: rectified linear unit activation function
- $hpool\ [s_f, s_t]$: Hanning window weighted averaged pooling operation with stride s_f in the frequency dimension and stride s_t in the time dimension
- $bnorm$: batch normalization operation
- $flatten$: multidimensional representation reshaped to a vector
- $fc\ [N]$: fully-connected layer with N units
- $dropout$: dropout regularization with 50% dropout rate

SUPPLEMENTARY REFERENCES

1. Hofman, P. M., Van Riswick, J. G. A. & Van Opstal, A. J. Relearning sound localization with new ears. *Nat. Neurosci.* **1**, 417–421 (1998).

2. Kulkarni, A. & Colburn, H. S. Role of spectral detail in sound-source localization. *Nature* **396**, 747–749 (1998).

3. Gardner, W. G. & Martin, K. D. HRTF measurements of a KEMAR. *J. Acoust. Soc. Am.* **97**, 3907–3908 (1995).

4. Grantham, D. W., Ricketts, T. A., Ashmead, D. H., Labadie, R. F. & Haynes, D. S. Localization by postlingually deafened adults fitted with a single cochlear implant. *The Laryngoscope* **118**, 145–151 (2008).

5. Bruce, I. C., Erfani, Y. & Zilany, M. S. A. A phenomenological model of the synapse between the inner hair cell and auditory nerve: Implications of limited neurotransmitter release sites. *Hear. Res.* **360**, 40–54 (2018).
