# Supplementary material for: Models optimized for real-world tasks reveal the task-dependent necessity of precise temporal coding in hearing
Source: Nat Commun. 2024 Dec 4;15:10590. doi: 10.1038/s41467-024-54700-5 (PMC11618365; doi:10.1038/s41467-024-54700-5)
Supplement: Supplementary file 2 — Reporting Summary [file 41467_2024_54700_MOESM2_ESM.pdf]

nature portfolio

Corresponding author(s): Mark R. Saddler; Josh H. McDermott

Last updated by author(s): Oct 30, 2024

Reporting Summary

Nature Portfolio wishes to improve the reproducibility of the work that we publish. This form provides structure for consistency and transparency in reporting. For further information on Nature Portfolio policies, see our Editorial Policies and the Editorial Policy Checklist.

Statistics

For all statistical analyses, confirm that the following items are present in the figure legend, table legend, main text, or Methods section.

n/a	Confirmed	
☐	☒	The exact sample size (n) for each experimental group/condition, given as a discrete number and unit of measurement
☐	☒	A statement on whether measurements were taken from distinct samples or whether the same sample was measured repeatedly
☐	☒	The statistical test(s) used AND whether they are one- or two-sided<br>*Only common tests should be described solely by name; describe more complex techniques in the Methods section.*
☒	☐	A description of all covariates tested
☐	☒	A description of any assumptions or corrections, such as tests of normality and adjustment for multiple comparisons
☐	☒	A full description of the statistical parameters including central tendency (e.g. means) or other basic estimates (e.g. regression coefficient) AND variation (e.g. standard deviation) or associated estimates of uncertainty (e.g. confidence intervals)
☐	☒	For null hypothesis testing, the test statistic (e.g. F, t, r) with confidence intervals, effect sizes, degrees of freedom and P value noted<br>*Give P values as exact values whenever suitable.*
☒	☐	For Bayesian analysis, information on the choice of priors and Markov chain Monte Carlo settings
☒	☐	For hierarchical and complex designs, identification of the appropriate level for tests and full reporting of outcomes
☐	☒	Estimates of effect sizes (e.g. Cohen's d, Pearson's r), indicating how they were calculated

Our web collection on statistics for biologists contains articles on many of the points above.

Software and code

Policy information about availability of computer code

Data collection	Downloaded publicly available audio datasets; computational modeling in Python 3.11.4, Tensorflow 2.13, and PyTorch 2.2.1; behavioral data collection via jsPsych 7.3 web browser experiments posted on the Prolific platform.
Data analysis	Python 3.11.4 packages: TensorFlow 2.13.0, PyTorch 2.2.1, Pandas 1.5.3, Numpy 1.24.3, Matplotlib 3.8.2 (Python environment to run code is included with the GitHub repository); Bruce, Erfani, Zilany (2018) Auditory Nerve Model (https://www.ece.mcmaster.ca/~ibruce/zbcANmodel/zbcANmodel.htm); STRAIGHT (https://github.com/HidekiKawahara/legacy_STRAIGHT).

For manuscripts utilizing custom algorithms or software that are central to the research but not yet described in published literature, software must be made available to editors and reviewers. We strongly encourage code deposition in a community repository (e.g. GitHub). See the Nature Portfolio guidelines for submitting code & software for further information.

Data

Policy information about availability of data

All manuscripts must include a data availability statement. This statement should provide the following information, where applicable:

- Accession codes, unique identifiers, or web links for publicly available datasets
- A description of any restrictions on data availability
- For clinical datasets or third party data, please ensure that the statement adheres to our policy

All data, models, and stimuli are available are available at https://github.com/msaddler/phaselocknet. Simulated auditory nerve representations of the sound datasets are prohibitively large to share; however, they can be generated with the provided code or made available upon request to the authors.

Research involving human participants, their data, or biological material

Policy information about studies with human participants or human data. See also policy information about sex, gender (identity/presentation), and sexual orientation and race, ethnicity and racism.

Reporting on sex and gender	Gender was self-reported by study participants. A gender-based analysis was not performed as we were interested in the comparison between humans and computational models and did not investigate individual differences in human behavior.
Reporting on race, ethnicity, or other socially relevant groupings	Race and ethnicity information was collected via voluntary self-report but was not considered in any analyses as we were interested in the comparison between humans and computational models and did not investigate individual differences in human behavior.
Population characteristics	See "Behavioural & social sciences study design"
Recruitment	Online participants were recruited on the Prolific platform with a geographic filter set to exclude individuals outside of the United States. Participants were invited to perform "word recognition in noise" and "celebrity voice recognition" studies. The United States filter was imposed because celebrity voice recognition trials in which participants self-reported being unfamiliar with the celebrity (in a survey before the experiment) could not provide usable data. United States-based participants were more likely to be familiar with the celebrity voices used. In-person participants for the sound localization in noise experiment were recruited from within the academic department.
Ethics oversight	The study was approved by the Committee on the Use of Humans as Experimental Subjects at MIT.

Note that full information on the approval of the study protocol must also be provided in the manuscript.

Field-specific reporting

Please select the one below that is the best fit for your research. If you are not sure, read the appropriate sections before making your selection.

☐ Life sciences ☒ Behavioural & social sciences ☐ Ecological, evolutionary & environmental sciences

For a reference copy of the document with all sections, see nature.com/documents/nr-reporting-summary-flat.pdf

Behavioural & social sciences study design

All studies must disclose on these points even when the disclosure is negative.

Study description	This quantitative study measured human abilities to recognize words in different noise conditions, recognize voices under different pitch manipulations, and localize sounds under different noise levels. Human data (online participants for the word and voice recognition experiments and in-person participants for the localization experiment) was averaged across participants and compared to model performance.
Research sample	For the word and voice recognition experiments, online participants were used for convenience (N=225). We screened for self-reported normal hearing and did not screen for age or self-reported gender. Based on our previous experience running online experiments, this sample was representative of typical online participant cohorts. For the in-person sound localization experiment, the 11 participants were MIT undergraduates, graduate students, and postdocs for convenience. The sample is representative of normal hearing humans experienced with listening experiments.
Sampling strategy	We used convenience sampling. As we were interested in obtaining measures of mean human performance to compare against model performance, sample sizes were increased until Spearman-Brown corrected split-half reliability was at least 0.9 for each experiment.
Data collection	Online participants completed word and voice recognition experiments in a web browser via surveys designed in jsPsych that played audio and recorded typed responses. In-person participants were played sounds from an array of loudspeakers and typed responses into a keypad.

Timing	Data were collected between June 2023 and December 2023.
Data exclusions	504 online participants were excluded from analyses for failing to pass the headphone check or performing poorly on independent catch trials. These were pre-established criteria meant to exclude participants that did not comply with online experiment instructions.
Non-participation	No in-person participant dropped out / declined participation. An unknown number of online participants on the Prolific platform dropped out, primarily due to technical difficulties (e.g., loss of internet connection or audio failing to play in the web browser). Participants could quit the experiments at any time without providing a reason.
Randomization	Participants were not allocated into experimental groups.

Reporting for specific materials, systems and methods

We require information from authors about some types of materials, experimental systems and methods used in many studies. Here, indicate whether each material, system or method listed is relevant to your study. If you are not sure if a list item applies to your research, read the appropriate section before selecting a response.

Materials & experimental systems

n/a	Involved in the study	
☒	☐	Antibodies
☒	☐	Eukaryotic cell lines
☒	☐	Palaeontology and archaeology
☒	☐	Animals and other organisms
☒	☐	Clinical data
☒	☐	Dual use research of concern
☒	☐	Plants

Methods

n/a	Involved in the study	
☒	☐	ChIP-seq
☒	☐	Flow cytometry
☒	☐	MRI-based neuroimaging

Plants

Seed stocks	*Report on the source of all seed stocks or other plant material used. If applicable, state the seed stock centre and catalogue number. If plant specimens were collected from the field, describe the collection location, date and sampling procedures.*
Novel plant genotypes	*Describe the methods by which all novel plant genotypes were produced. This includes those generated by transgenic approaches, gene editing, chemical/radiation-based mutagenesis and hybridization. For transgenic lines, describe the transformation method, the number of independent lines analyzed and the generation upon which experiments were performed. For gene-edited lines, describe the editor used, the endogenous sequence targeted for editing, the targeting guide RNA sequence (if applicable) and how the editor was applied.*
Authentication	*Describe any authentication procedures for each seed stock used or novel genotype generated. Describe any experiments used to assess the effect of a mutation and, where applicable, how potential secondary effects (e.g. second site T-DNA insertions, mosiacism, off-target gene editing) were examined.*
